# Minor Salivary Gland Polymorphous Adenocarcinoma With Local Recurrence After Seven Years: A Case Report

**DOI:** 10.7759/cureus.40112

**Published:** 2023-06-08

**Authors:** Luiz Antônio R Santos, Márcio Tadeu V Brito

**Affiliations:** 1 Radiology, Instituto Nacional de Câncer, Rio de Janeiro, BRA

**Keywords:** magnetic resonance imaging (mri), computed tomography (ct ), head and neck cancer, imaging diagnosis, salivary gland tumors, minor salivary gland

## Abstract

Polymorphous adenocarcinoma is a rare neoplasm of the minor salivary glands with an indolent behavior. Here, we report and describe the computed tomography (CT) and magnetic resonance imaging (MRI) of polymorphic adenocarcinoma in a 69-year-old patient who presented with local recurrence seven years after the initial treatment. On contrast CT, the primary lesion appeared heterogeneous and invaded the pterygopalatine fossa and the sphenopalatine foramen. The recurrent lesion on MRI showed a hypointense signal on the T1-weighted sequence, a hyperintense signal on the T2-weighted sequence, and heterogeneous enhancement with contrast. The patient underwent a new surgery for lesion resection and is currently under clinical and radiological follow-up. It is recommended to follow patients for at least 15 years after diagnosis, as local recurrences can occur up to 10 years after the initial treatment.

## Introduction

Salivary gland tumors are rare, accounting for approximately 8% of head and neck tumors [1]. Polymorphous adenocarcinoma (PCA) represents the third most common oral malignant tumor of the minor salivary glands [2]. PCA has characteristics that can mimic benign pathologies, such as slow growth, which can make early diagnosis a challenge [3]. Here, we present the case of a 69-year-old female patient diagnosed with PCA and recurrence seven years after the initial treatment along with a description of the radiological findings.

## Case presentation

**Figure 1:**
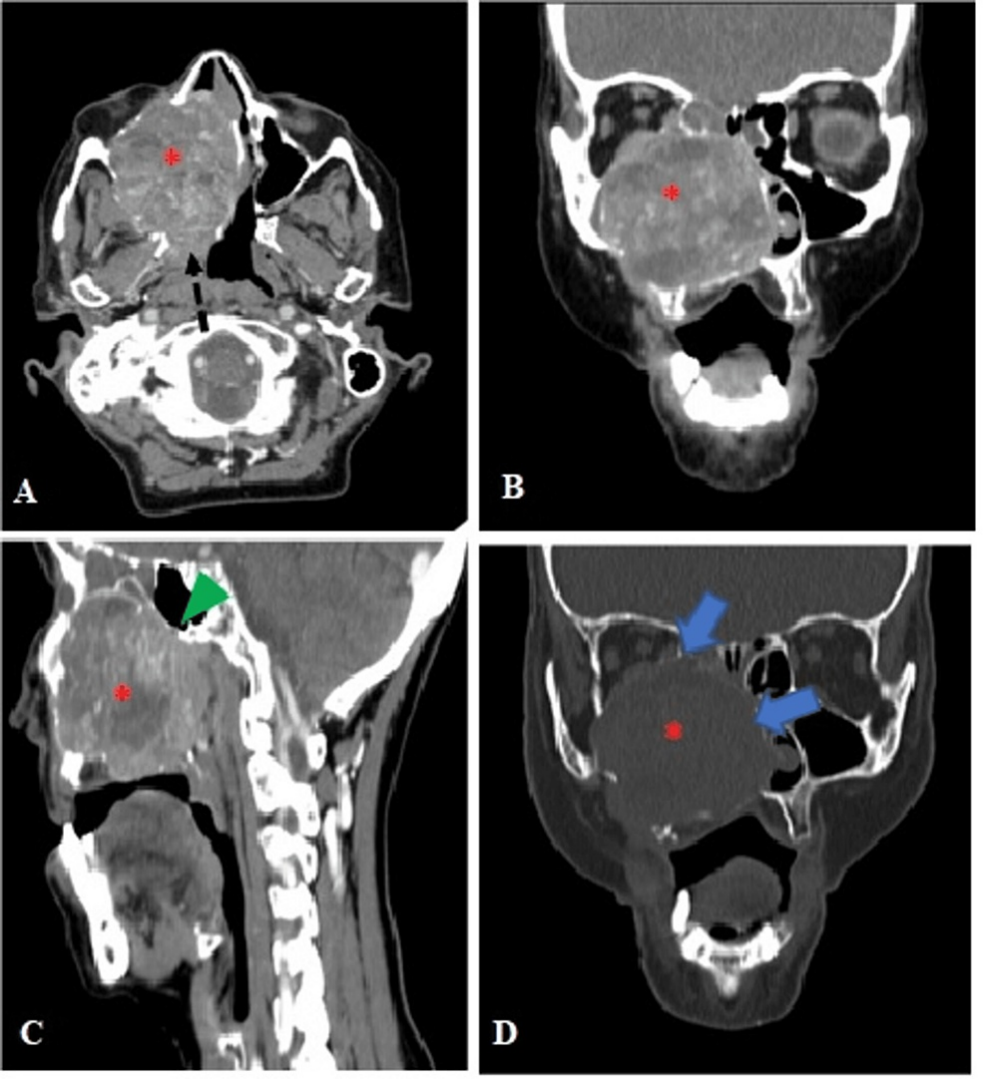
A 66-year-old patient with a mass in the right maxilla. Contrast CT of the head and neck showing an expansile lesion with heterogeneous contrast enhancement in the right maxillary sinus (asterisk) (A-D), infiltration of the pterygopalatine fossa (dashed arrow) (A), and the sphenopalatine foramen (arrowhead) (C). The bone window CT shows bone remodeling (arrow) (D).

A 69-year-old female patient presented with an enlargement in the right maxillary region associated with pain and reduced mouth opening. The symptoms had progressively worsened over the last five months. The patient denied smoking and alcoholism. During the physical examination, a fixed mass was palpated in the region of the right maxilla. Computed tomography (CT) of the neck showed a large solid expansive mass without calcifications inside, with heterogeneous contrast enhancement. The mass compromised the maxillary sinus, maxilla, and right nasal fossa, with diffuse bone remodeling and multiple small areas of cortical ruptures. The mass was well delimited, with numerous hypodense, irregular areas of varying sizes that insinuated into the lower thirds of the orbit, the ethmoid, and the roof of the oral cavity to the right and the left nasal cavity. The lesion infiltrated the pterygopalatine fossa and sphenopalatine foramen, destroyed the pterygoid plates on that side, and bulged the rhinopharyngeal wall in contiguity, with a blurring of the tubal torus (Figure 1), with no evidence of distant disease. Among the diagnostic radiological possibilities, cystic adenoid carcinoma was suggested. When performing the biopsy of the lesion, the histopathological result was PAC originating in the minor salivary gland.

**Figure 2:**
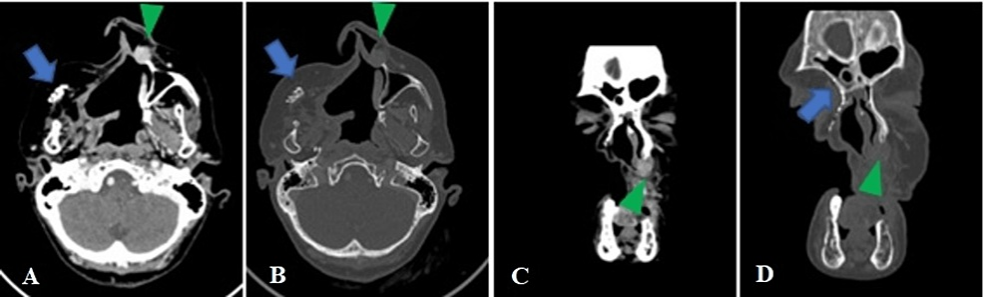
Seven years after the initial treatment. Contrast-enhanced CT after extended maxillectomy (A-D), with reconstruction (arrow). Circumscribed nodular lesion (arrowhead) located in the left nasal septum.

**Figure 3:**
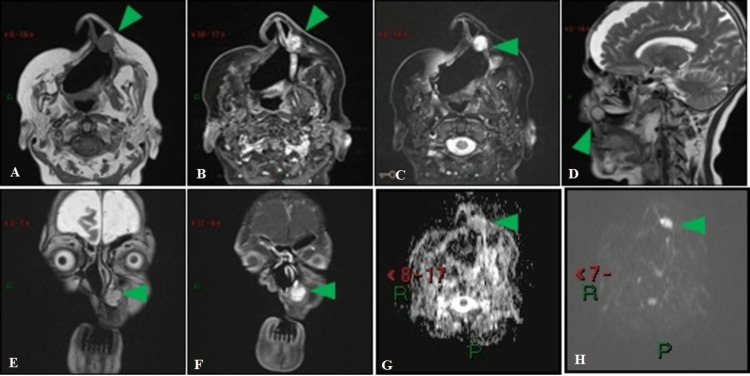
MRI seven years after the initial treatment. Contrast-enhanced MRI after extended maxillectomy, with reconstruction. Pre-contrast image (A) shows a lesion (arrowhead) with T1 hyposignal and heterogeneous contrast medium impregnation on post-contrast images (B, F). The lesion showed hyperintensity on T2 fat-suppressed and T2-weighted image (C-E) and low diffusion restriction (G-H). b-value in diffusion-weighted images = 1.000.

The patient underwent a total right maxillectomy, and histopathological analysis of the specimen confirmed the diagnosis of PAC originating in the minor salivary gland. The tumor had infiltrated connective tissue, bone, and perineural tissue, but no vascular invasion was observed. The patient remained under follow-up once a year for seven years until she complained of pain in the infranasal and left maxillary region. CT (Figure 2) and magnetic resonance imaging (MRI) (Figure 3) of the neck showed a circumscribed nodular lesion measuring 1.5 × 1.4 × 1.5 cm, which involved the left frontal process of the maxilla, maintaining contact with the cartilaginous portion of the nasal septum and the inferior turbinate. The lesion showed a hypointense signal on T1-weighted and a hyperintense signal on T2-weighted sequences, with heterogeneous contrast enhancement and mild diffusion restriction. Physical examination revealed a vegetating lesion located in the inferior nasal turbinate and left nasal septum in its medial component, measuring approximately 2.0 cm in length. Biopsy results confirmed the recurrence of the primary tumor, and the patient underwent a new tumor resection with free margins. The patient remains under clinical/radiological follow-up with a complete physical examination of the oral and nasal cavity twice a year and an MRI of the face annually.

## Discussion

Salivary glands are exocrine structures responsible for producing and secreting saliva and are divided into two groups, namely, major and minor. The major salivary glands are the parotid, submandibular, and sublingual glands. The minor salivary glands are acini that are distributed in the mucosa of the oral cavity and pharynx, mainly in the transition between the hard and soft palate. The control of salivary secretion of the minor salivary gland is carried out by the pterygopalatine ganglion, optic ganglion, and submandibular ganglion [4].

Salivary gland tumors are a rare and heterogeneous group of lesions, accounting for approximately 2.6%-8% of head and neck tumors in adults [1,5,6]. PAC, previously classified as polymorphic low-grade adenocarcinoma [2], is the third most common oral malignant tumor, representing 0.4%-2.4% of all salivary gland tumors and occurring mainly on the hard palate [7]. PAC predominantly affects women, at a ratio of 3:1, between the third and fourth decades of life [8]. It has an indolent course, with slow growth and presenting symptoms mainly related to lesion growth. Local and distant metastases are considered rare, with cervical and lung lymph nodes being the most frequently affected sites, respectively [9].

PAC presents with cytological uniformity, morphological diversity, and an infiltrative growth pattern; stains positive for S100 on pathology; has *PRKD *gene rearrangements; and may have a target appearance on histopathology [10].

Lesions originating from minor salivary glands require imaging examinations to confirm their presence and determine their characteristics and extension [9]. PAC imaging findings are nonspecific. On CT, PAC may have thin, irregular walls with heterogeneous contrast enhancement. Furthermore, CT provides information on bone invasion by the lesion [3,11,12]. On MRI, the lesions may appear as an intermediate signal on T1 and hyperintense on T2 [13], with low values (0.95 ± 0.09) on the apparent diffusion coefficient map [14]. MRI also provides information about perineural lesion invasion [15]. These imaging features are also described in adenoid cystic carcinoma.

In the radiological investigation of salivary gland tumors, other differential diagnoses should be considered. Among benign pathologies, pleomorphic adenoma stands out, which on MRI shows a hypointense signal on T1-weighted sequences, a hyperintense signal on T2-weighted sequences, and usually exhibits homogeneous enhancement. In the group of malignant neoplasms, the differential diagnosis should be made with adenoid cystic carcinoma. On imaging, adenoid cystic carcinoma presents as an intermediate-to-low signal on both T1 and T2-weighted sequences. Mucoepidermoid carcinoma often demonstrates a low-to-intermediate signal on both T1 and T2 imaging, with cystic components appearing more T2 hyperintense. Acinic cell carcinoma shows a hyperintense signal on T2-weighted sequences for cystic/necrotic components, and the solid component exhibits a mildly hyperintense signal on T2-weighted sequences [10].

Surgery is the preferred treatment, with complete excision of the lesion. In the presence of bone invasion of the palate, maxillectomy is performed to a variable extent, with immediate reconstruction to maintain functionality or after a disease-free period. Lymph node dissection can be performed if alterations in lymph nodes are identified on imaging examinations [16]. Radiotherapy is used when there is no complete resection of the lesion, the surgical margins are not disease-free, there is perineural or perivascular invasion, or when lymph node disease is present [17,18].

Follow-up of patients can be routinely performed with clinical examinations and MRI [19] for a period of 15 years [20]. Local recurrence occurs between 5% and 33% of cases within five to ten years. Low-dose chest CT should also be performed to assess metastases [9].

## Conclusions

This report describes the radiological and pathological features of a rare case of PAC with metastasis occurring seven years after primary tumor resection. It also highlights that the radiological diagnosis can be challenging and may be confused with other more common tumors because it presents with low signal on T1-weighted sequences, hyperintensity on T2-weighted images, and homogeneous enhancement on post-contrast T1-weighted MRI images, as well as with heterogeneous contrast enhancement on CT. Furthermore, it emphasizes that follow-up of patients with this neoplasm should be conducted with regular clinical and radiological examinations for the diagnosis of local recurrence for a minimum period of 15 years.
